# Human Mesenchymal Stem Cells Suppress the Stretch–Induced Inflammatory miR-155 and Cytokines in Bronchial Epithelial Cells

**DOI:** 10.1371/journal.pone.0071342

**Published:** 2013-08-13

**Authors:** Yi-Chun Kuo, Yi-Shuan Julie Li, Jing Zhou, Yu-Ru Vernon Shih, Marina Miller, David Broide, Oscar Kuang-Sheng Lee, Shu Chien

**Affiliations:** 1 Institute of Clinical Medicine, National Yang-Ming University, Taipei, Taiwan; 2 Department of Bioengineering and Institute of Engineering in Medicine, University of California San Diego, La Jolla, California, United States of America; 3 Department of Medicine, University of California San Diego, La Jolla, California, United States of America; 4 Department of Orthopaedics and Traumatology, Taipei Veterans General Hospital, Taipei, Taiwan; 5 Stem Cell Research Center, National Yang-Ming University, Taipei, Taiwan; H.Lee Moffitt Cancer Center & Research Institute, United States of America

## Abstract

Current research in pulmonary pathology has focused on inflammatory reactions initiated by immunological responses to allergens and irritants. In addition to these biochemical stimuli, physical forces also play an important role in regulating the structure, function, and metabolism of the lung. Hyperstretch of lung tissues can contribute to the inflammatory responses in asthma, but the mechanisms of mechanically induced inflammation in the lung remain unclear. Our results demonstrate that excessive stretch increased the secretion of inflammatory cytokines by human bronchial epithelial cells (hBECs), including IL-8. This increase of IL-8 secretion was due to an elevated microRNA-155 (miR-155) expression, which caused the suppression of Src homology 2 domain–containing inositol 5-phosphatase 1 (SHIP1) production and the subsequent activation of JNK signaling. *In vivo* studies in our asthmatic mouse model also showed such changes in miR-155, IL-8, and SHIP1 expressions that reflect inflammatory responses. Co-culture with human mesenchymal stem cells (hMSCs) reversed the stretch-induced hBEC inflammatory responses as a result of IL-10 secretion by hMSCs to down-regulate miR-155 expression in hBECs. In summary, we have demonstrated that mechanical stretch modulates the homeostasis of the hBEC secretome involving miR-155 and that hMSCs can be used as a potential therapeutic approach to reverse bronchial epithelial inflammation in asthma.

## Introduction

Physical forces, including shear stress and stretching force, influence the structure, function and metabolism of lung cells [1], [2], [3]. Cells in the respiratory airway are constantly exposed to mechanical stretches due to cyclic expansions and deflations of the lung. The lungs in asthma patients with increased respiration rates experience greater stretch beyond that during normal respiration [4], [5]. Mechanical stretches regulate airway remodeling, and the high pressures associated with enhanced ventilation *in* *vivo* have been shown to modulate airway gene expression [6], [7]. It has been demonstrated that mechanical stretch *in* *vitro* regulates epithelial signaling [8], gene expression [8], and pulmonary functions [9], [10]. It has also been shown that asthma attacks can trigger deep inspirations with increases in amplitude and frequency and that the resulting hyperstretch tends to worsen the airway obstruction [11], [12]. *Ex vivo* hyperstretching (2.5×of basal tone) of human bronchi isolated from patients can cause myogenic and pulmonary inflammatory responses (e.g., epithelial release of leukotrienes) [13]. These studies suggest that hyperstretch play an important role in modulating pulmonary homeostasis.

Emerging information has revealed miRs as critical regulators of gene expression and hence many cellular functions in health and disease. Since miRs regulate 30% of human gene expression [14], the miR expression signatures can be employed as biomarkers for tissue functions and diseases. In airway cells, miR expression profiles can be regulated by multiple factors, including growth factors [15], inflammatory agents [16], mechanical force [17], and hypoxia [18]. miRs have been demonstrated to play critical roles in many inflammatory diseases and asthma [19], [20], but the roles of miRs in regulating the mechanical pathobiology of the lung remain to be established.

Human mesenchymal stem cells (hMSCs) are multipotent adult stem cells which were first discovered in bone marrow stroma as fibroblast-like non-hematopoietic stem cells [21]. These cells have the capacity to differentiate into multiple lineages, including osteoblasts, adipocytes, endothelial cells, myocytes, astrocytes, and hepatocytes [22], [23], [24], [25], [26]. hMSCs have been shown to play an important role in modulating the immune system and proposed to be a potential therapeutic modality for many clinical conditions, including inflammatory diseases [27], [28], [29], [30], [31]. Thus, hMSCs can be considered for usage as immunomodulators to rescue hyperstretch-induced inflammation in hBECs.

We have studied the mechanism by which hyperstretch induces hBEC inflammatory responses by utilizing an *in* *vitro* stretch system [32] with a frequency of 40 cycles/min (cpm) and an amplitude of 10% to mimic the *in* *vivo* pathological states [33]. We demonstrated that hyperstretch induces miR-155 to reduce SHIP1, leading to the activation of JNK and the consequential IL-8 secretion. The results were confirmed with the use of an OVA-induced asthmatic mouse model [34]. The *in* *vivo* studies on bronchoalveolar lavage and lung tissues from the asthmatic mice showed increased expressions of IL-8 and miR-155, and decreased expressions of IL-10 and SHIP1; these *in* *vivo* pulmonary inflammatory reactions are in agreement with the findings on hBECs under hyperstretch *in* *vitro.* Furthermore, we have shown that hMSCs can mitigate the miR-155-mediated inflammatory responses due to hyperstretch by using a hMSC/hBEC co-culture stretch system.

## Materials and Methods

### Cell Culture

Human bronchial epithelial cells (hBECs; S9, ATCC CRL-2778) alone, or with human bone marrow-derived mesenchymal stem cells (hMSCs; Lonza), were grown in high-glucose DMEM (Invitrogen) supplemented with 10% FBS and 1% penicillin–streptomycin at 37°C and plated on silicon sheets (0.25 mm thickness), assembled in a stretch chamber [32] placed in a cell-culture incubator at 37°C and atmosphere of 5% CO_2_.

### Stretching Experiments

We have created a device that generates equibiaxial stretch [32]. In each stretch chamber, a silicone membrane is secured to a polycarbonate holder by using a silicone rubber O-ring and positioned over a Teflon indenter. The cells were seeded onto fibronectin-coated stretch chambers, at a concentration of 40,000 cells per chamber with a surface area of 2×2 cm^2^. The chambers were assembled onto a mobile plate attached to a cam, which was rotated by a DC motor to generate a vertical sinusoidal displacement against the indenters, thus cyclically deforming the membranes. Stretch amplitude and frequency were controlled by varying the mobile plate displacement and motor speed. The hyperstretch experiments were performed with 10% area increase at a frequency of 40 cpm to mimic the *in* *vivo* pathological condition. Cells were incubated for 24 hours before the stretching experiments. Non-stretched cells culturing in the static chambers were used as controls.

### Real-Time Polymerase Chain Reaction (RT-PCR)

Total RNA was isolated from 3×10^5^ cells using Trizol (Invitrogen). Four or more biological repeats were performed for all miR and RNA expression studies. PCR was performed with miR-specific primers for TaqMan miR assays (Applied Biosystems) according to manufacturer’s protocol. The relative expression levels of miRs in cells were normalized by internal controls and determined with ΔΔCT. The specific primer sequences are 5′-ACCGGAAGGAACCATCTCACT-3′ (forward) and 5′-GGAAGGCTGCCAAGAGAGC-3′ (reverse) for IL-8; 5′-TGCCTTCAGCAGAGTGAAGA-3′ (forward) and 5′-GCAACCCAGGTAACCCTTAAA-3′ (reverse) for IL-10; 5′-AGCCACATCGCTCAGACAC-3′ (forward) and 5′-GCCCAATACGACCAAATCC-3′ (reverse) for GAPDH.

### Cytokine Array

Mouse and human cytokine Multi-Analyte ELISArray Kit (SABiosciences) were used to measure the cytokine production of IL1A, IL1B, IL2, IL4, IL5, IL6, IL8, IL10, IL12, IL13, IL17A and Granulocyte-Macrophage Colony Stimulating Factor (GM-CSF) in mouse BAL with or without OVA-treated, or supernatants of conditioned media of hBECs with or without stretch after 24 hours of culture. The arrays were performed according to the manufacturer’s instructions. The absorbance levels of the cytokines were measured on a plate reader (Beckman Coulter DTX 880) at 450 nm. Sampling was performed in triplicates including standards. 12 arrays were used for each human and mouse experiment. Standard errors of the mean were calculated from three biological repeats.

### OVA-induced Asthmatic Mouse Model

BALB/c mice were challenged with OVA over a period of 21–28 days as described previously [35], [36]. In brief, the animals were sensitized by intraperitoneal injection of OVA (20 µg/100 µl), followed by intranasal challenge with 1% OVA in PBS every other day for 3–4 weeks. The OVA-induced responses were assessed by determining the cell counts and protein concentration in post-mortem BAL fluid, and measuring the cytokine and IgE levels in serum [35,35]. All animal procedures were approved by the University of California San Diego Institutional Animal Care and Use Committee, and met guidelines of the National Institutes of Health.

### Immunohistology Staining

The expression levels of SHIP and miR-155 in lung tissues were detected by immunostaining with a specific antibody for SHIP1 (Santa Cruz Biotechnology) and a specific probe, miRCURY LNA™ Detection probe, 250 pmol, 5`-DIG and 3`-DIG labeled, for mouse miR-155 (EXIQON). Negative control experiments omitting primary antibody and probe were included for each experiment (data not shown).

### ELISA Assay

Conditioned media collected from stretch experiments or static controls were centrifuged to remove cell debris, and the volumes of the various samples were determined. The IL-8 and IL-10 concentrations in the media were measured with R&D ELISA kits. Briefly, 100 µl of the media were applied to the immunoplate precoated with anti-human or anti-mouse monoclonal antibody. Secondary detection antibody of each assay was then added to the immobilized IL-8 and IL-10 in the sample. The conjugation of anti- IL-8 and IL10 with their antigens was visualized using Avidin-HRP substrate. The arrays were performed according to the manufacturer’s instructions. The absorbance levels of proteins were measured on a plate reader at 450 nm. Sampling was performed in triplicates.

### Antibodies and Reagents

Mouse monoclonal antibody (mAb) against p-AKT was purchased from Cell Signaling Technology. Rabbit polyclonal antibodies against p-JNK, c-Jun and SHIP1, and mouse monoclonal antibodies against β-actin and AKT were obtained from Santa Cruz Biotechnology. The chemical inhibitors SP 600125 and LY 294002 (purchased from EMD Millipore Chemical) were used at the concentration of 20 µM to block the activities of JNK and PI3K-AKT, respectively. Anti-miR-155, Pre-miR155, and the respective negative control inhibitor and mimic were purchased from Ambion. The siRNA for SHIP1 and control sequences were purchased from Santa Cruz Biotechnology. The SHIP1 and control overexpression plasmids were purchased from InvivoGen.

### Statistical Analysis

Statistical analysis was performed by Student’s t test for two groups of data and by one-way ANOVA for multiple comparisons. Data are expressed as mean ± SEM from three independent experiments. P<0.05 was considered statistically significant.

## Results

### Asthmatic Challenges Induce Inflammatory Cytokines in Mouse Lungs

**Figure 1:**
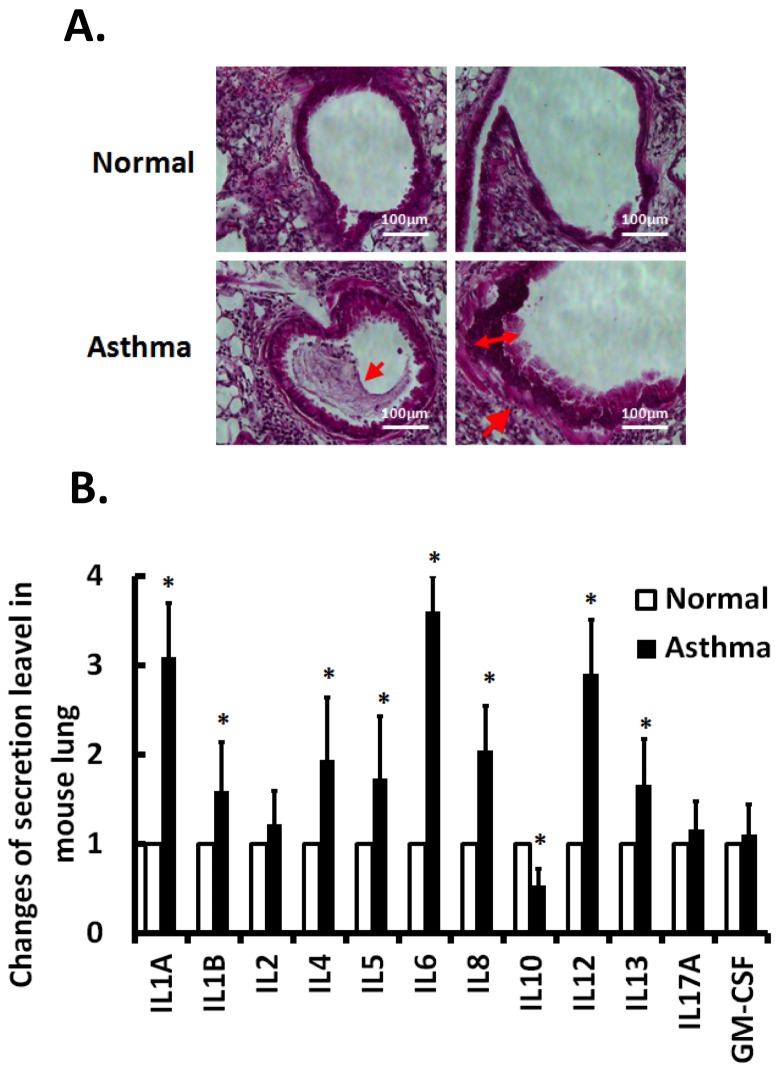
Inflammatory responses in OVA-induced asthmatic mouse model. (A) Bronchial tissue specimens of normal and asthma mice were prepared and stained with hematoxylin and eosin (H&E). Presence of mucus plug and basal membrane thickening in bronchial was found in asthmatic mice (lower panel), but in the normal lung (top panel). (B) Cytokines expressions were analyzed using the mouse cytokine Multi-Analyte ELISArray. The samples from asthmatic mice were normalized to ones from normal mice. Data represent mean ± s.e.m. *, P<0.05; n = 3.

Using a mouse cytokine Multi-Analyte ELISArray Kit (SABiosciences), we demonstrated that mice challenged with ovalbumin (OVA) exhibited significant increases of the pro-inflammatory cytokines interleukin 1A, 1B, 4, 5, 6, 8, 12 and 13, and decreases of the anti-inflammatory cytokine interleukin 10 (IL-10) in their bronchoalveolar lavage (BAL) (Fig. 1A). In addition, the airways of these asthmatic mice demonstrated the general features of inflammatory reactions, including the appearance of edematous submucosa, thickening of the basal membrane, increase in epithelial permeability, and recruitment of immune cells to the lung tissue (Fig. 1B). These results indicate the modulations of interleukins in asthmatic responses.

### Hyperstretch Modulates Cytokines Secretion in hBECs and hMSCs

**Figure 2:**
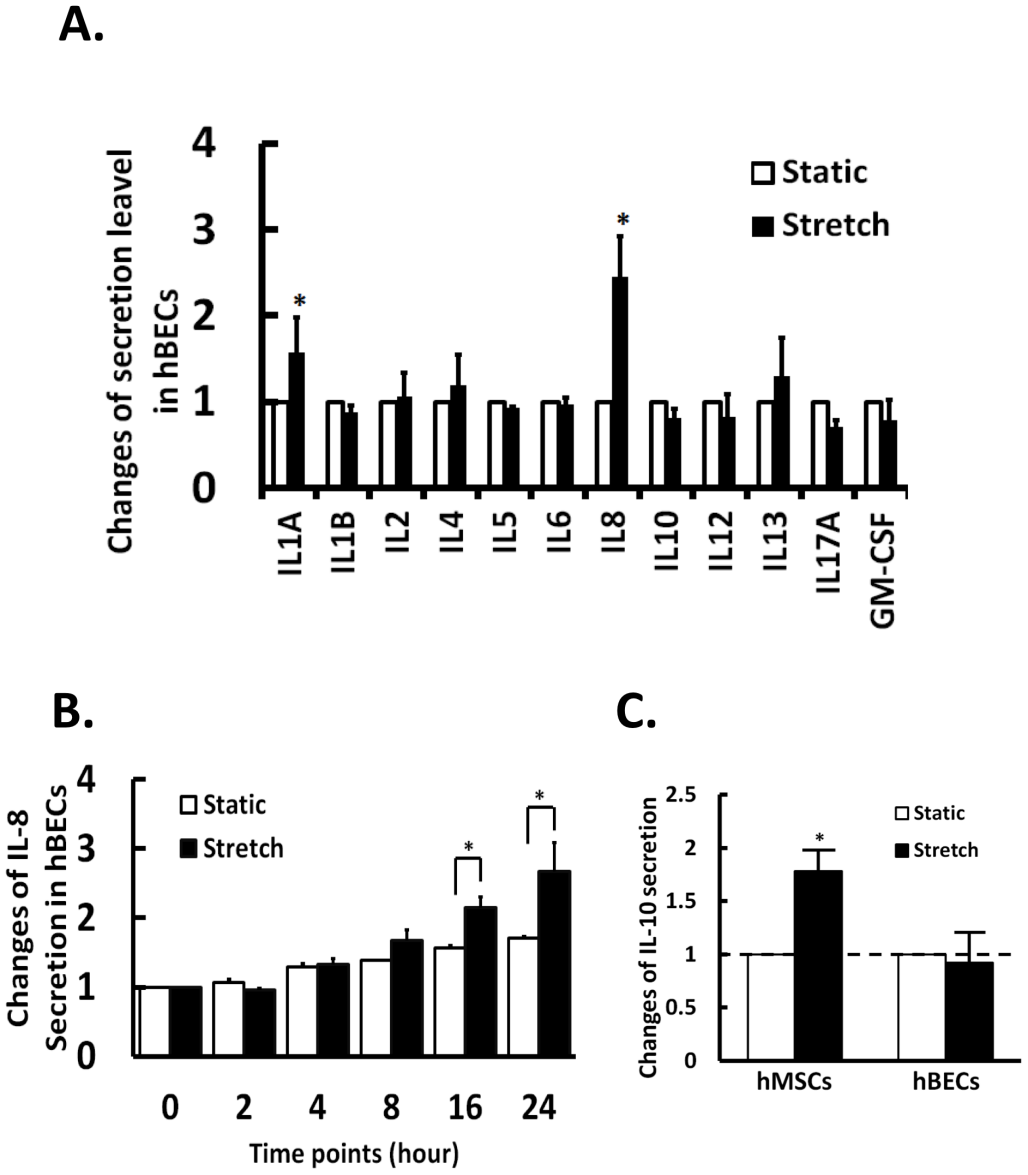
Secretion of inflammation-related cytokines by hBECs under hyperstretch. (A) Cytokine levels in conditioned medium of hBEC under hyperstretch were measured using a human cytokine Multi-Analyst ELISA array and normalized to the conditioned medium of static control. (B) Hyperstretch regulation of pro-inflammatory IL-8 secretion in hBECs over 24 hour period of time. (C) Hyperstretch regulation of anti-inflammatory cytokine IL-10 secretion by hBECs and hMSCs. The changes in secretion level are relative to static control. Data represent mean ± s.e.m. *, P<0.05; n = 3.

It has been shown that hyperstretching human bronchi causes myogenic and inflammatory responses [38]. Our human cytokine array results indicate that the secretions of the inflammatory IL-1A and IL-8 increased significantly in hBECs under the hyperstretch condition of 40 cycles/min and 10% strain (Fig. 2A). Measurement of IL-8 levels in hBECs with PCR and ELISA showed that the stretch-induced increases of IL-8 mRNA expression (Fig. S1, left panel) and secretion (Fig 2B) was a time-dependency process during the 24-hour period of study. It is well known that hMSCs serve as excellent immunomodulators both *in* *vitro* and *in* *vivo* by secreting various cytokines and growth factors, including the anti-inflammatory IL-10, to regulate the immune system during inflammation [39], [40], [41]. Therefore, we tested the regulation of IL-10 expression and secretion in hMSCs and hBECs by hyperstretch. In hMSCs, hyperstretch significantly increased the IL-10 mRNA expression (Fig. S2 right panel) and secretion (Fig. 2C right panel), but had little effect on IL-8 (Fig. S2, left panel). In hBECs, however, hyperstretch decreased IL-10 mRNA expression (Fig. S1 right panel), but did not significantly alter the IL-10 secretion (Fig. 2C left panel). These results demonstrate that hyperstretch of hBECs induces pro-inflammatory and decreases anti-inflammatory cytokines. In contrast, hyperstretch of hMSCs causes the increase of the anti-inflammatory cytokine secretion. These findings suggest that hMSCs have a high potential for suppression of inflammation and may potentially rescue the hyperstretch-induced inflammation in hBECs.

### hMSCs Suppress Hyperstretch-induced IL-8 Secretion in hBECs via the Paracrine Effect of IL-10

**Figure 3:**
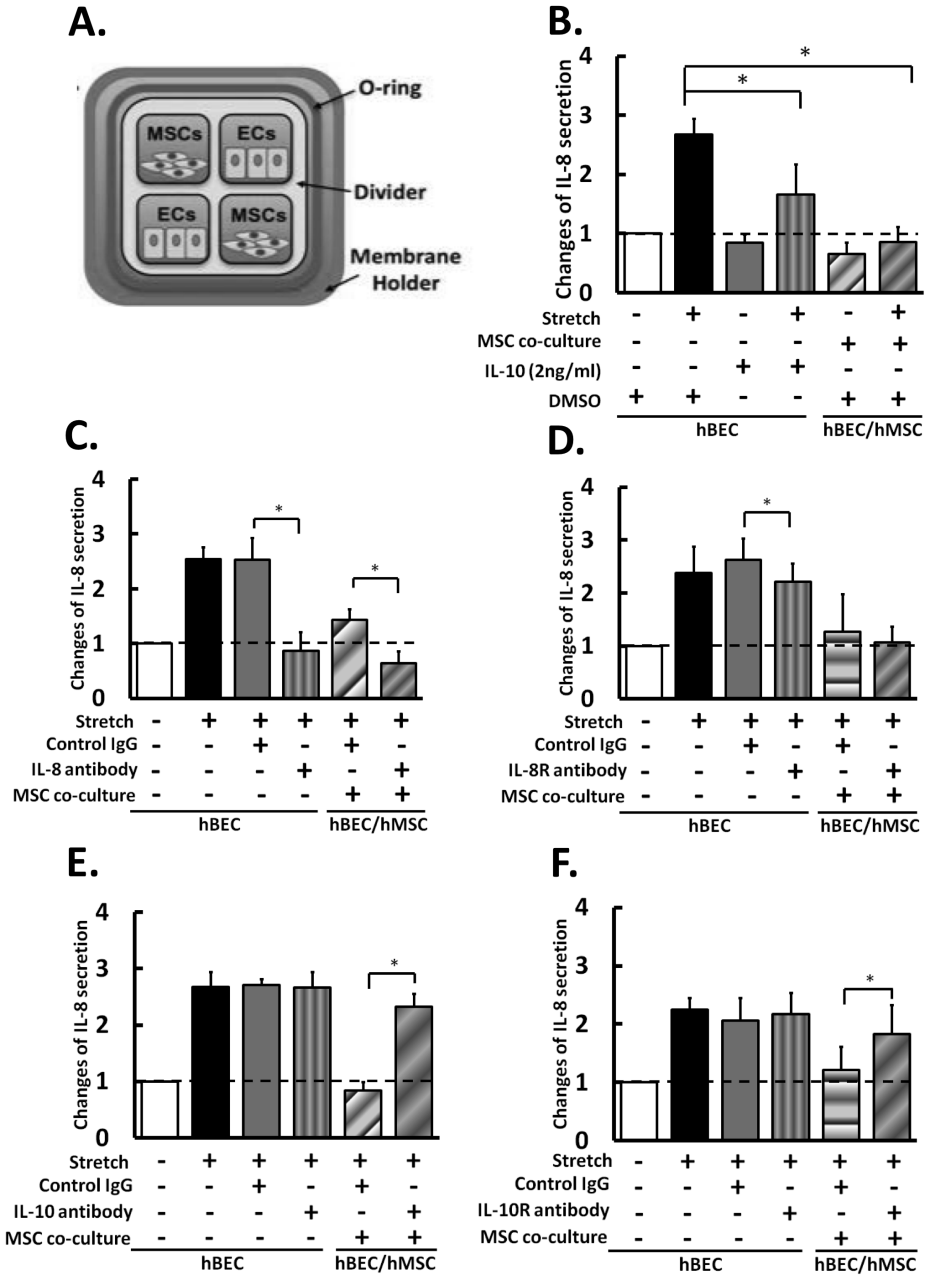
The roles of IL-8 and IL-10 in hyperstretch-induced IL-8 secretion in hBEC/hMSC co-culture. (A) Schematic drawing of the top view of the stretching co-culture system. Measurement of IL-8 secretion levels in hBECs (first four bars) and in hBEC/hMSC (5^th^ and 6^th^ bar) after treatments with (B) exogenous IL-10, (C) IL-8 antibodies, (D) IL-8 receptor [IL-8R] blocking antibodies, (E) IL-10 antibodies, and (F) IL-10 receptor [IL-10R] blocking antibodies. The results demonstrated that hMSCs and IL-10 had similar effects, whereas IL-8 and hMSCs had contrary effects, on hyperstretch-induction of hBEC IL-8 secretion. The changes in secretion level are relative to static control. Data represent mean ± s.e.m. *, P<0.05; **, P<0.01 n = 5.

We have used a co-culture system to investigate the interaction between hMSCs and hBECs under hyperstretch (Fig. 3A). In the monocultured hBECs, IL-10 treatment suppressed the IL-8 secretion (Fig. 3B). The IL-8 secretion by hBECs was not significantly altered by co-culture with hMSCs under static condition (Fig. 3B, bars 1 and 5), but was significantly reduced under hyperstretch condition (Fig. 3B, bars 2 and 6). In addition, the hyperstretch-induced hBEC IL-8 secretion can be significantly reduced by the conditioned media from stretched (Fig. S3), but not unstretched (data not shown), hMSCs. The effect, however, is less than that found in direct hMSC co-culture, indicating that the proximity of interaction between hBECs and hMSCs is important for the regulation of inflammatory homeostasis under hyperstretch. Together with the result that hyperstretch induces hMSC IL-10 secretion (Fig. 2C), our findings suggest that hMSCs may become effective for immunosupression under hyperstretch. The blocking antibodies for IL-8, IL-10, and their respective receptors were used to decipher their roles in hMSC-hBEC interaction under hyperstretch. Blocking the IL-8 in the culture medium (Fig. 3C) or the hBEC IL-8 receptor (IL-8R) (Fig. 3D) reduced the hyperstretch-induced IL-8 secretion by mono-cultured hBECs. Blocking IL-8, but not IL-8R, also enhanced the suppressive effect of hMSCs on IL-8 secretion by the co-cultured hBECs. In contrast, blocking IL-10 in the culture medium (Fig. 3E) or the hBEC IL-10 receptor (IL-10R) (Fig. 3F) abrogated the suppressive effect of hMSCs on IL-8 secretion by the co-cultured hBECs. These results indicate that hMSCs suppress hyperstretch-induced inflammation in hBECs via an increased secretion of IL-10, which inhibits IL-8 secretion by hBECs via a paracrine mechanism.

### Hyperstretch Increases mirna-155 Production in hBECs and the Reversal of this Effect by Co-culture with hMSCs

**Figure 4:**
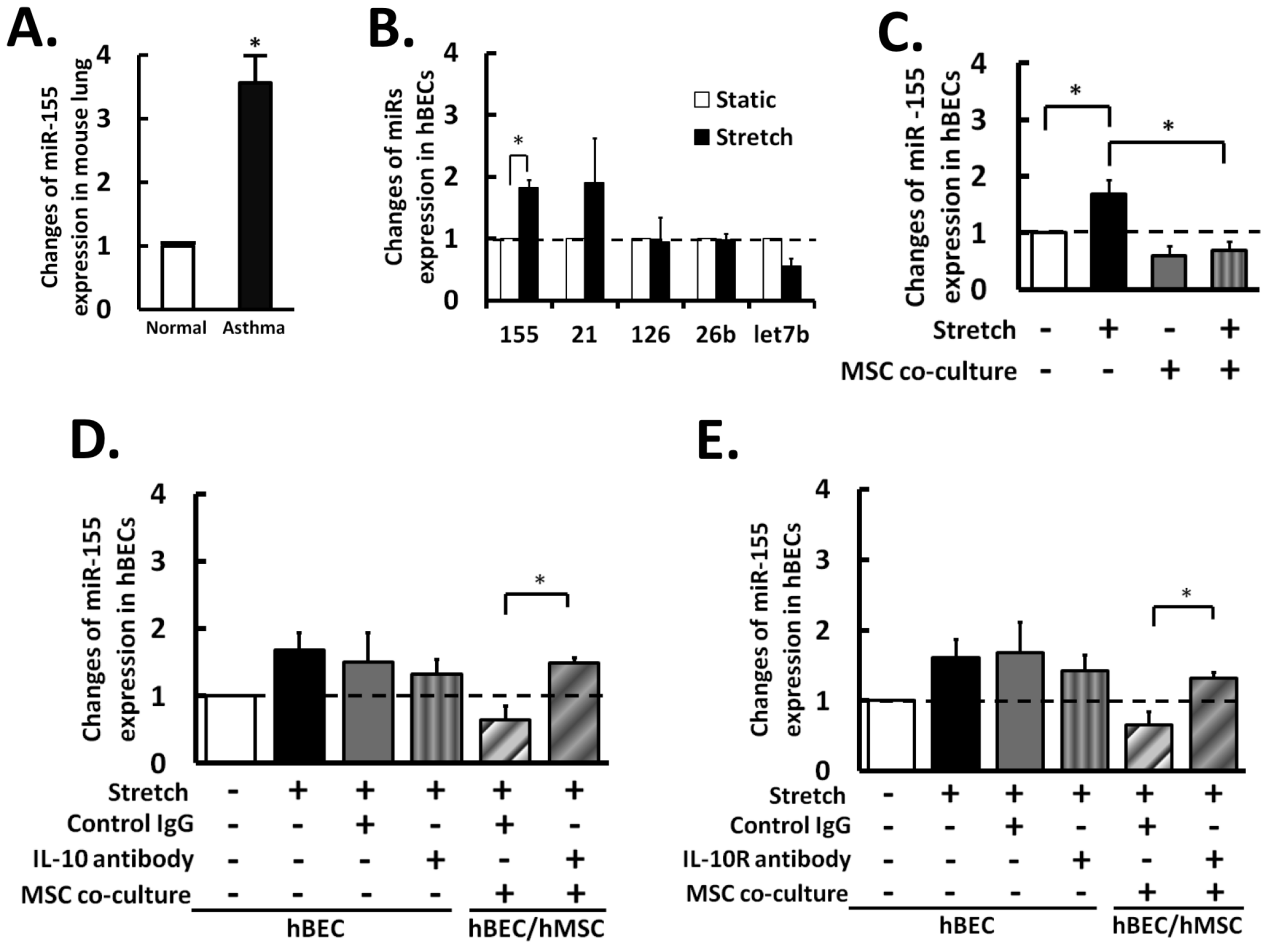
The miRs expression in cells under inflammatory challenages. (A) The expression of miR-155 in asthmatic and normal lung tissues of mice. (B) Examination of hyperstretch-regulated miRs, where miR-155 was significantly increased. (C) Hyperstretch regulation of miR-155 in hBECs with or without hMSCs co-culture. Expression levels of hBEC miR-155 in mono-culture (first four bars), and in co-culture (5^th^ and 6^th^ bar) after treatments with (D) IL-10 antibodies, and (E) IL-10 receptor [IL-10R] blocking antibodies. The results demonstrated that hMSCs and IL-10 had similar effects on up-regulation of hyperstretch-induction miR-155 expression in hBEC. The changes in secretion level are relative to static control. Data represent mean ± s.e.m. *, P<0.05; **, P<0.01 n = 5.

miRs have been demonstrated to play critical roles in many inflammatory diseases including asthma [19], [20], but the roles of miR in mechanical stretch of hBECs and in mechano-pathobiology of the lung remain to be determined. We found that miR-155 was highly expressed in asthmatic mouse lung tissues compared to non-asthmatic normal tissues (Fig. 4A). We further screened inflammatory miRs in hBECs that respond to hyperstretch, and identified miR-155 as being significantly increased by hyperstretch (Fig. 4B).

The hyperstretch-induced miR-155 expression level in mono-cultured hBECs was abolished by co-culturing with hMSCs (Fig. 4C) and addition of exogenous IL-10 (Fig. S4), suggesting that miR-155 may play a role in mediating the hyperstretch-induced hBEC-hMSC interaction. Blocking IL-10 (Fig. 4D), and IL-10R (Fig. 4E) also reduced the suppressive effect of hMSCs on miR-155 expression. These results indicate that hMSCs suppress hyperstretch-induced inflammation in hBECs via an increased secretion of IL-10, which inhibits the expression of miR-155 by hBECs via a paracrine mechanism.

### MiRNA-155 Modulates IL-8 and IL-10 Secretion in hBECs and hMSCs

**Figure 5:**
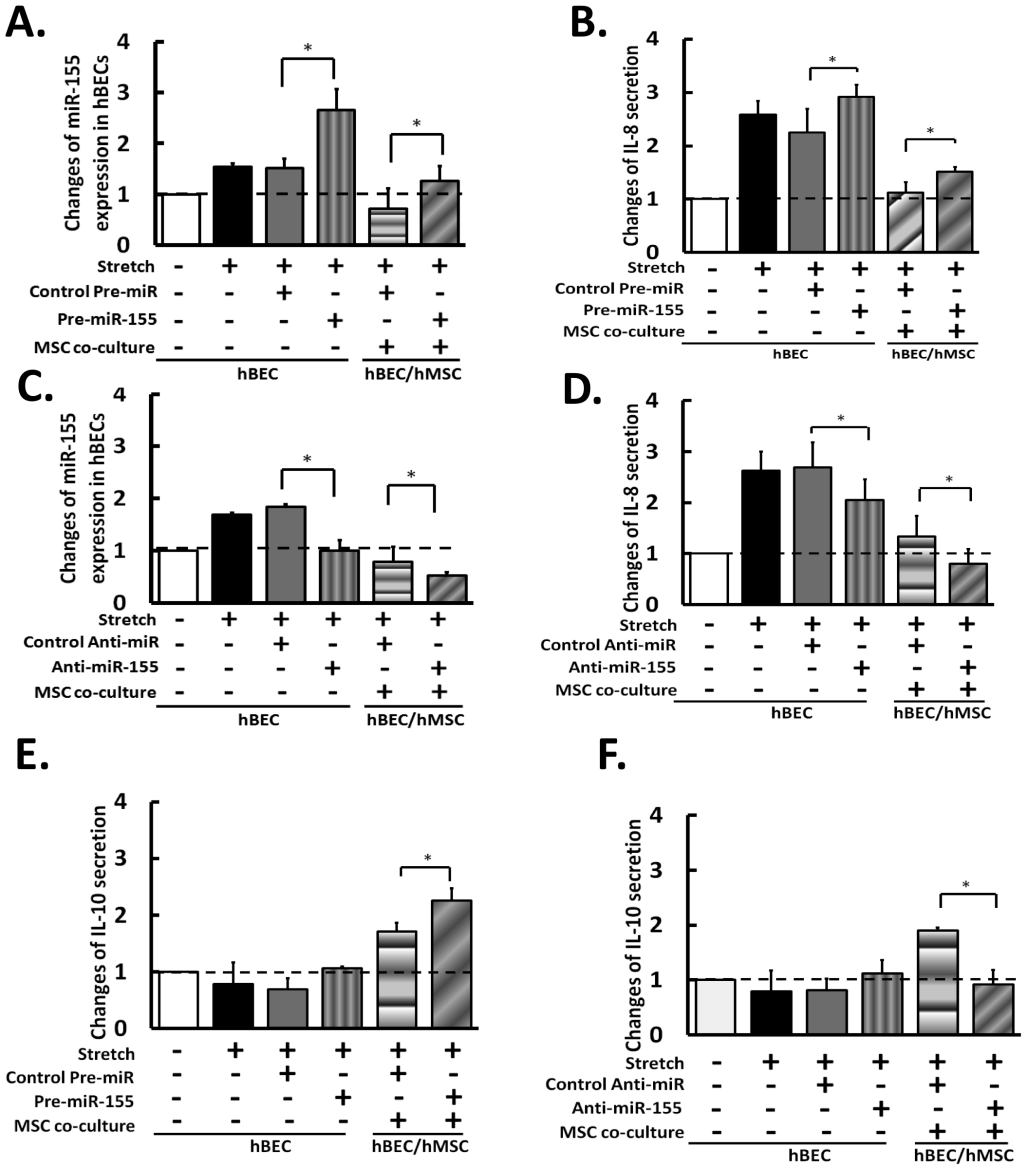
The role of miR-155 in hyperstretch-regulation of hBEC-hMSC inflammatory responses. The effects of overexpression of miR-155 on (A) miR-155 levels and (B) IL-8 secretion in hBECs/hMSC co-culture under hyperstrech. The effects of anti-miR-155 on (C) miR-155 levels and (D) IL-8 secretion in hBECs/hMSC co-culture under hyperstrech. The effects of (E) overexpression of miR-155 and (F) anti-miR-155 on IL-10 secretion. In (D)–(F), the measurement of IL-8/10 secretion levels were done either in mono-culture (first four bars) and co-culture (5^th^ and 6^th^ bar) after treatments with pre-miR and anti-miR. The changes in cytokines secretion and mRNA expression are relative to static control. Data represent mean ± s.e.m. *, P<0.05; n = 5.

To investigate the role of miR-155 in regulating hBEC inflammatory responses, we introduced pre-miR-155 and anti-miR-155 to alter the miR expression level in hBECs. The transfection of pre-miR-155 further increased the levels of miR155 expression (Fig. 5A) and IL-8 secretion (Fig. 5B) in hyperstretched hBECs in mono-culture. Pre-miR-155 also reversed the suppression of miR-155 and IL-8 in hyperstretched hBECs by co-culture with hMSC (Figs. 5A and 5B). Anti-miR-155 abolished the hyperstretch-induced miR-155 expression and attenuated IL-8 secretion in mono-cultured hBECs, and enhanced the suppressive effects of hMSCs on co-cultured hBECs (Figs. 5C and 5D). In hBEC mono-culture, pre- and anti-miR-155 had little effects on IL-10 secretion. In hBEC/hMSC co-culture, however, the hMSC-induced IL-10 secretion is enhanced by the increase of miR-155 with pre-miR155 (Fig. 5E) and abolished by the knockdown of miR-155 with ant-miR155 (Fig. 5F). These results suggest that miR-155 may also be involved in the anti-inflammation pathways in hBEC/hMSC interactions.

### miRNA-155 Mediates hBECs Inflammation through SHIP1

**Figure 6:**
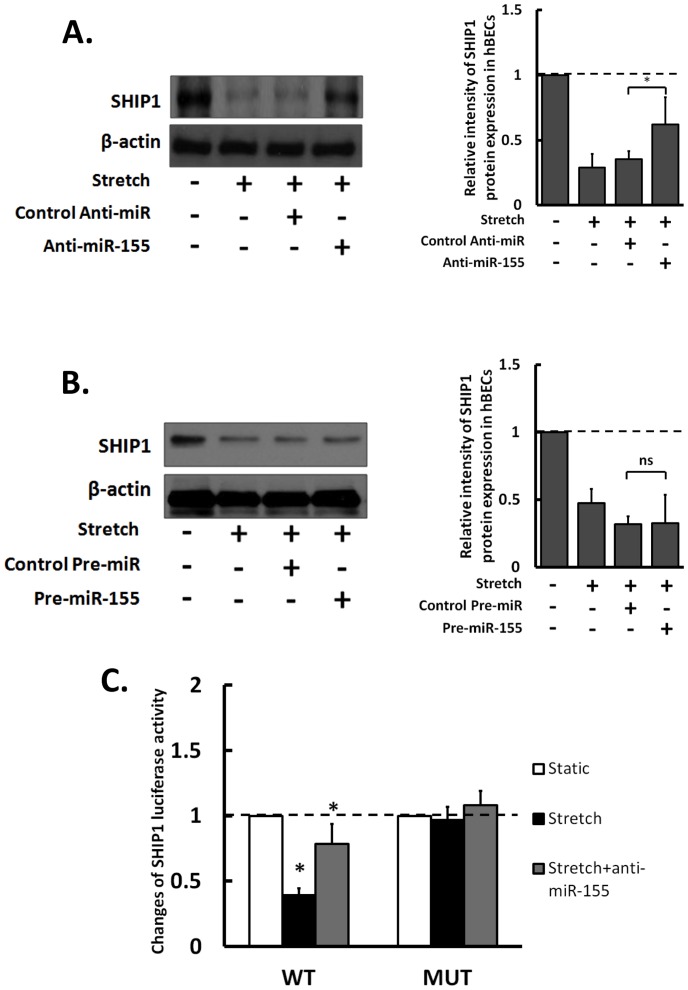
MiR-155 targeting SHIP1 under hyperstretch. Stretch-reduction of SHIP1 is reversed by anti-miR-155 (A), but not pre-miR-155 (C), compared with non-specific control anti-miR and control pre-miR, respectively. Relative intensity of SHIP1 protein expression was normalized to the expression ofβ-actin and static control. (C) Determination of SHIP1 is a direct target for miR-155 by using SHIP1 3′-UTR luciferase activity with/without anti-miR-155 treatment under hyperstretch. The changes in luciferase activity are relative to static control. Data represent mean ± s.e.m. *, P<0.05; n = 5.

The above results showed that miR-155 is involved in the hyperstretch-induced inflammation by modulating the responses in hBECs. However, the detailed mechanism by which miR-155 mediates hBECs inflammation remains unknown. Previous studies have shown that SHIP1 is a potential target during inflammation [42], [43]. We first tested the role of miR-155 in hyperstretch-regulation of SHIP1 expression. Knockdown miR-155 with anti-miR-155 attenuated the hyperstrech-suppression of SHIP1 levels (Fig. 6A). Overexpression of miR-155 with pre-miR-155 caused a decrease of SHIP1 level in hBECs under static condition (data not shown), but it had little effect on the stretch-reduction of SHIP1 (Fig. 6B). Using hSHIP1 3′UTR luciferase constructs with wild type (WT) or mutant (MUT) miR-155 target sequences, we demonstrated that hyperstretch down-regulated WT, but not the mutant, hSHIP1 3′UTR luciferase in hBECs. The addition of anti-miR-155 abolished the hyperstretch-suppression of WT-hSHIP1 3′UTR-luciferase activity (Fig. 6C). These results suggest that miR-155 directly targeted SHIP1 to regulate hBEC signaling under hyperstretch.

### Overexpression of SHIP1 in hBECs Reversed the Stretch-induced miR-155 Level

**Figure 7:**
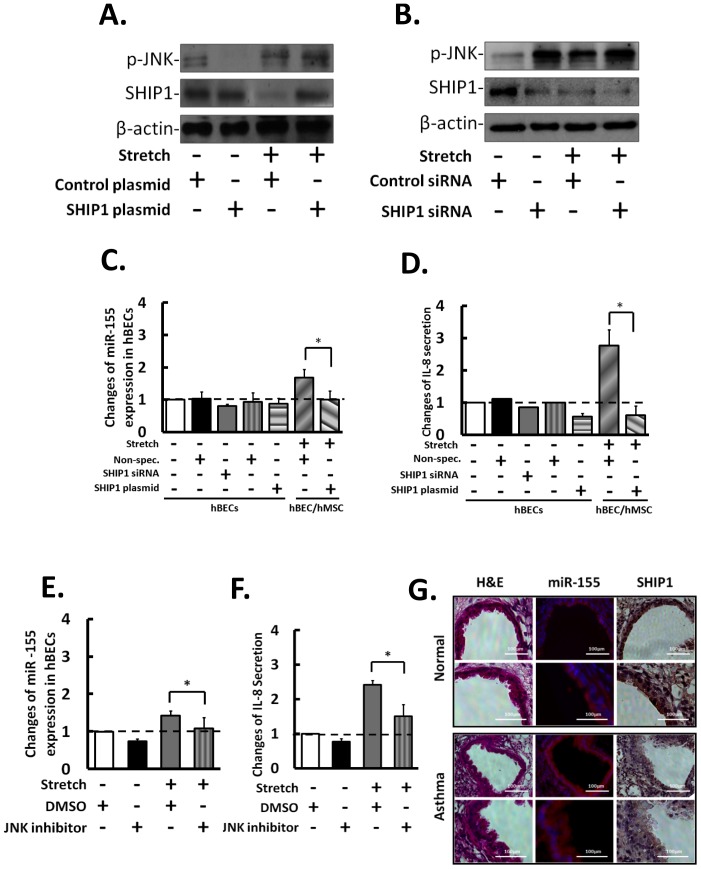
The role of SHIP1 in hBEC signaling and inflammation. (A) Overexpression of SHIP1 attenuated the hyperstretch-induced JNK phosphorylation. (B) SHIP1 knockdown caused increases of JNK phosphorylation under static condition, but not on stretch inducibility. (C) and (D) Overexpresion SHIP1 attenuated the hyperstretch-induced miR-155 and IL-8 secretion compared with non-specific (non-spec.) plasmid control. Knocking down SHIP1 had no significant effect compare with non-specific (non-spec.) siRNA control under static condition. Inhibition of SHIP1 downstream JNK attenuated the (E) miR-155 expression and (F) IL-8 secretion. (G) Confirmation of the elevation of miR-155 expression and low level of SHIP1 asthmatic lung tissues. The changes in cytokines secretion and mRNA expression are relative to static control. Data represent mean ± s.e.m. *, P<0.05; n = 5.

SHIP1 has been shown to be an upstream regulator for MAPK [44] and PI3K [45], [46], [47] pathways. We investigated the miR-155/SHIP1 downstream signaling events that lead to hyperstretch-regulated signaling events by overexpressing and knocking down the endogenous SHIP1 in hBECs under hyperstretch. Overexpression of SHIP1 rescued the hyperstretch-suppression of SHIP1, and therefore, attenuated the stretch-induced inflammatory signaling activation, including the phosphorylations of JNK (Fig. 7A) and AKT (Fig. S5A). Knockdown SHIP1 with siRNA increased the phosphorylations of JNK and AKT under static condition, but not under the hyperstretch condition (Figs. 7B and S5B) in which the SHIP1 level was already low (Fig. 7B). The functional role of hyperstretch-regulated SHIP1 in hBEC inflammation was also confirmed by testing the effects of SHIP1 modulation on hBEC IL-8 secretion and miR155 expression. Knockdown SHIP1 in hBECs with siRNA had little effect on miR-155 expression (Fig. 7C) and IL-8 secretion (Fig. 7D), but SHIP1 overexpression negatively regulated miR-155 expression (Fig. 7C) and IL-8 secretion (Fig. 7D) in hBECs under hyperstretch. The inhibition of JNK led to the reductions of hBEC miR-155 expression (Fig. 7E) and IL-8 secretion (Fig. 7F), but these effects are not seen with AKT inhibition (Fig. S6), suggesting that miR155-SHIP1-JNK may play an important role in the hyperstretch-induced hBEC inflammatory responses. Based on these *in* *vitro* observations, we examined the expression of miR-155 and SHIP1 in lung tissues of normal and asthmatic mice *in* *vivo.* Indeed, the miR-155 expression level was elevated in asthmatic samples (Fig. 7G), and the SHIP1 expression was significantly lower than in the asthmatic samples than normal control. Thus, our results indicate that miR-155 expression is significantly induced under hyperstretch in asthmatic lung to repress SHIP1 expression and induce the inflammatory signaling in hBECs. The finding that the expression of miR-155 can be attenuated by SHIP1 overexpression suggests a potential therapeutic role of SHIP1 in mitigating pulmonary inflammation.

## Discussion

Asthma is a disease of airway dysfunction that involves epithelial damages, bronchoalveolar lavage fluid mediator accumulation, increased cytokine secretion, and epithelial hyperplasia [48]. There is increasing evidence that mechanical forces, including stretching, act on lung cells to affect their structure, function, metabolism, and signaling [1], [2], [3], [8], [9], [10]. In asthma patients with increased respiration rates, the lung is subjected to stretch beyond that during normal respiration [4], [5], and the hyperstretch in asthma attacks tends to worsen the airway obstructions [11], [12] via myogenic and pulmonary inflammatory responses [13]. The acute responses (within 5 min) of hyperstretch-induced human bronchi inflammation involves NOS activation, and Rho, Wnt signaling [13]. However, the impact of long-term hyperstretch, as in asthmatic lung, on pulmonary responses remains undetermined. In the current study we investigated the mechanisms by which 24-hour hyperstretch regulates pulmonary cell inflammatory reactions. Our *in* *vitro* studies on cultured hBECs subjected to 24-hour hyperstretch and *in* *vivo* studies on bronchoalveolar lavage from 3–4 week OVA-induced asthmatic mice have shown increased expressions of pro-inflammatory cytokines such as IL-8 and decreased expressions of the anti-inflammatory cytokine IL-10 (Figs. 1 and 2). Our results demonstrated that asthmatic lung significantly increases the secretion of multiple interleukins, including IL-1A, 1B, 4, 5, 6, 8, 12, 13 (Fig. 1B), while *in* *vitro* hyperstretch only significantly induced IL1A and 8. The discrepancy of the results may be partially due to the difference in experimental duration, i.e., the asthmatic mice were under challenge for 3–4 weeks, while hyperstretch only lasted for 24 hr. In addition, it is likely that the *in* *vivo* environment involves multiple cell types which may modulate the responses, while in vitro hyperstretch only acted on the epithelial cells. Nevertheless, the *in* *vitro* hyperstretch system has provided the evidence that hyperstretch induces pulmonary cell inflammatory responses within 24 hours. Such a system allows us to further decipher the molecular mechanisms of hyperstretch responses of hBECs, as well as their interactions with hMSCs.

hMSCs have been recognized as an excellent immune moderator both *in* *vivo* [35], [49], [50] and *in* *vitro* [51], [52] by secreting a repertoire of anti-inflammatory cytokines such as IL-10 to suppress inflammation [49] in response to various mechanical forces [53], [54]. We modified the *in* *vitro* co-culture stretch camber (Fig. 3A) to study the interactions between hMSCs and hBECs under hyperstretch. Our results demonstrated that the hyperstretch-induced IL-8 secretion by hBECs can be suppressed by co-culture with hMSCs and by using the conditioned media from stretched hMSCs. The suppression effect was abrogated by blocking IL-10 in the culture medium or the IL-10R on hBECs and enhanced by blocking IL-8 or IL-8R, These findings indicate that hMSCs suppress hyperstretch-induced inflammation in hBECs via a paracrine mechanism.

MiRs act as critical regulators of gene expression and cellular functions in health and disease. A recent study on profiling miR expression in asthmatic mice demonstrated the elevated levels of multiple miRs, including the ones related to inflammatory responses [55]. In screening inflammatory miRs in hBECs that respond to hyperstretch, we identified miR-155 is significantly increased by hyperstretch (Fig. 4B). The hyperstretch-induced miR-155 expression, as the IL-8 secretion, in mono-cultured hBECs was abolished by co-culturing with hMSCs. Figs. 3E and 3F showed that the hMSCs suppression of hBECs inflammation is mediated by IL-10. Recent study showed that IL-10 inhibits LPS-induced miR-155 expression [56], [57]. The study supports our results that IL-10 mediates the hMSC suppressive effect on hBEC inflammation via inhibition of miR-155. We also found that the hyperstretch-inductions of miR155 and IL-8 in hBECs were enhanced by pre-miR-155 and mitigated by anti-miR-155 (Fig. 4). In hBECs, the secretion level of IL-10 was not affected by pre-miR-155 and anti-miR-155 (Fig. 5E and 5F). In hMSCs, however, the IL-10 secretion was significantly up-regulated by pre-miR-155 and down-regulated by anti-miR-155, indicating that miR-155 augments IL-10 production by hMSCs during hyperstretch-induced inflammation Together with the finding that IL-10 mediates the hMSC suppressive effect via inhibition of miR-155 (Figs. S4, 4, and 5), these results suggest a negative feedback in which an increase of miR-155 would stimulate the release of IL-10 to decrease miR-155 and hence the inflammatory response. However, the mechanism by which hyperstretch induces miR-155 expression remain unclear. Recent studies suggest that expression of miR-155 in cancer growth and inflammation can be regulated by a variety of receptors and transcription factors, including TLR4 and AP-1 [58] and that IL-10 inhibits miR-155 by blocking the TLR4 signaling [59]. Mechanical stretch has been shown to modulate the activities of those molecules [60]. Further investigations are needed to further eluidate the molecular mechanism by which hyperstretch regulates IL-10/miR-155 signaling in hBEC inflammation.

MiR-155 is involved in many important biological processes [61], and it has been identified to play a critical role in modulating the crosstalk in cancer and inflammation [62]. More importantly, miR-155 has been shown to be a potential pro-inflammatory regulator in lung diseases [63]. Src homology 2 domain–containing inositol 5-phosphatase 1 (SHIP-1) is a primary target of miR-155 during inflammation [42], [43], we tested the role of miR-155 in hyperstretch regulation of SHIP1 expression. SHIP1 expression was down-regulated by hyperstretch, and this was reversed by anti-miR-155 in hBECs. SHIP1 overexpression negatively regulated miR-155 expression and IL-8 secretion in hBECs under hyperstretch *in* *vitro*. The induction of miR-155 and reduction of SHIP1 in lung tissues were confirmed in asthmatic mouse over normal controls *in* *vivo* (Fig. 7E), which is similar to the responses of miR-155 and SHIP1 in hBECs under hyperstretch *in* *vitro*. These results indicate that miR-155 represses SHIP1 expression and induces the inflammatory signaling both *in* *vitro* and *in* *vivo*. Furthermore, the finding that overexpression of SHIP1 attenuated hyperstretch-induction of miR-155 suggests a potential feedback mechanism for an anti-inflammatory role of SHIP1. Further investigations are needed to establish the interaction between SHIP1 and miR-155 in greater detail.

**Figure 8:**
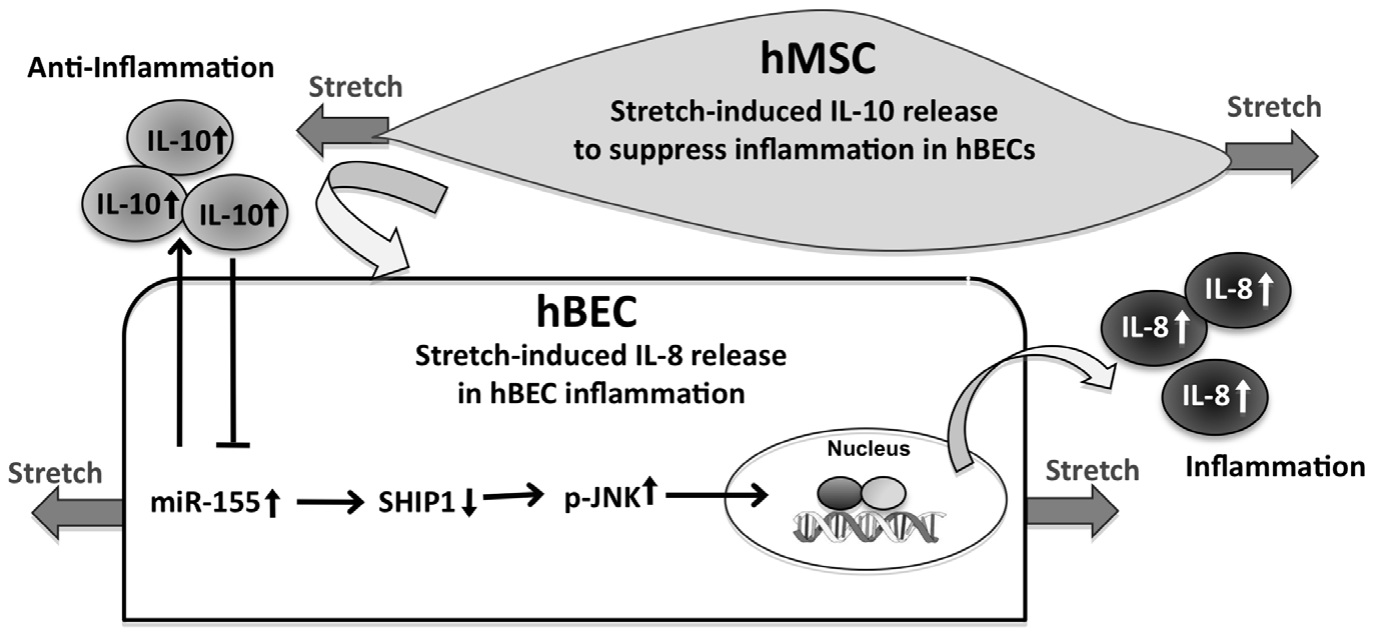
Schematic representation of the role of hyperstretch and hMSC in pulmonary cell inflammation. Hyperstretch induces miR-155 to target SHIP1, which leads to the increases of JNK activation and consequential IL-8 secretion. Co-culture of hMSCs exhibits the anti-inflammation effects to reverse the hyperstretch-induced hBEC inflammatory responses.

It has been shown that hyperstretch induced epithelial IL-8 expression through the JNK/NFκB pathway [64], and that miR-155 suppresses SHIP to activate AKT pathway to promote inflammation in lung epithelial cells [65]. Consistent with these findings in the literature, our results demonstrate that hyperstretch induces miR155 to suppress SHIP1, thus leading to the activation of JNK and AKT pathways (Figs. 7A, 7B, and Fig. S5). In addition, the addition of exogenous IL-10 attenuated the stretch-induced JNK and AKT phosphorylation, while overexpression of pre-miR155 induced JNK and AKT phosphorylation (data not shown). However, only the inhibition of JNK (Fig. 7), but not AKT (Fig. S6), attenuated the hyperstretch-induced inflammation in hBEC. In summary, our results indicate that hMSC co-culture suppresses the hyperstretch-induced inflammation by secreting IL-10 to inhibit the miR-155-SHIP-JNK signaling (Fig. 8). Although hyperstretch is not the only factor that causes inflammation in the asthmatic mouse model, our data shed new insights on how miR-155 mediates the hMSC-regulation of inflammation and reveal the therapeutic potentials of hMSCs and miR-155 for the treatment of asthma and related lung diseases.

The current study, by integrating biomechanics, vascular biology, and systems biology approaches, have contributed to the understanding of the roles of hMSCs and miR-155 in regulating hBEC functions in response to hyperstretch. We have established the molecular and mechanical bases of hyperstretch-regulation of miR-155 in mono-cultured hBECs and hBEC/hMSC co-culture, and the consequent gene modulation and functional manifestation. For the first time, we successfully developed a mechanical stretch co-culture platform to investigate the interactions between different cell types under stretch condition. This study has elucidated the molecular basis of hBEC/hMSC interaction under hyperstretch that is relevant to the pathophysiology of asthma.

## Supporting Information

Figure S1 **Hyperstretch regulation of IL-8 and IL-10** **mRNA levels in hBECs.** Data represent mean ± s.e.m. *, P<0.05; n = 3.
(TIF)

Figure S2 **Hyperstretch regulation of IL-8 and IL-10** **mRNA levels in hMSCs.** Data represent mean ± s.e.m. *, P<0.05; n = 3.
(TIF)

Figure S3 **The effects of hMSCs co-cultured or hMSCs condition medium (CM) on hBEC IL-8 secretion under hyperstretch.** Data represent mean ± s.e.m. *, P<0.05; n = 3.
(TIF)

Figure S4 **The effects of exogenous IL-10 treatment on miR-155 expression under hyperstretch.** Data represent mean ± s.e.m. *, P<0.05; n = 3.
(TIF)

Figure S5 **The effects of SHIP1 overexpression or knockdown on AKT phosphorylation under hyperstretch.** Data represent mean ± s.e.m. *, P<0.05; n = 3.
(TIF)

Figure S6 **The effects of AKT inhibition on miR-155 expression and IL-8 secretion under hyperstretch.** Data represent mean ± s.e.m. *, P<0.05; n = 3.
(TIF)

## References

[1] Radell P, Jonzon A (1992) Afferent activity in pulmonary stretch receptors before and after lung injury. Ups J Med Sci 97: 127–139. 1471312 10.3109/03009739209179289

[2] Russo LA, Rannels SR, Laslow KS, Rannels DE (1989) Stretch-related changes in lung cAMP after partial pneumonectomy. Am J Physiol 257: E261–268. 2548393 10.1152/ajpendo.1989.257.2.E261

[3] Manny J, Grindlinger G, Mathe AA, Hechtman HB (1978) Positive end-expiratory pressure, lung stretch, and decreased myocardial contractility. Surgery 84: 127–133. 351839

[4] Kumar A, Lnu S, Malya R, Barron D, Moore J, et al (2003) Mechanical stretch activates nuclear factor-kappaB, activator protein-1, and mitogen-activated protein kinases in lung parenchyma: implications in asthma. Faseb Journal 17: 1800–1811. 14519659 10.1096/fj.02-1148com

[5] Koller EA, Ferrer P (1973) Discharge patterns of the lung stretch receptors and activation of deflation fibres in anaphylactic bronchial asthma. Respir Physiol 17: 113–126. 4688282 10.1016/0034-5687(73)90114-x

[6] Tschumperlin DJ, Drazen JM (2006) Chronic effects of mechanical force on airways. Annu Rev Physiol 68: 563–583. 16460284 10.1146/annurev.physiol.68.072304.113102

[7] Uhlig S (2002) Ventilation-induced lung injury and mechanotransduction: stretching it too far? Am J Physiol Lung Cell Mol Physiol 282: L892–896. 11943651 10.1152/ajplung.00124.2001

[8] Copland IB, Post M (2007) Stretch-activated signaling pathways responsible for early response gene expression in fetal lung epithelial cells. J Cell Physiol 210: 133–143. 16998809 10.1002/jcp.20840

[9] Bai Y, Bai X, Wang Z, Zhang X, Ruan C, et al (2011) MicroRNA-126 inhibits ischemia-induced retinal neovascularization via regulating angiogenic growth factors. Exp Mol Pathol 91: 471–477. 21586283 10.1016/j.yexmp.2011.04.016

[10] Crosby LM, Luellen C, Zhang Z, Tague LL, Sinclair SE, et al (2011) Balance of life and death in alveolar epithelial type II cells: proliferation, apoptosis, and the effects of cyclic stretch on wound healing. Am J Physiol Lung Cell Mol Physiol 301: L536–546. 21724858 10.1152/ajplung.00371.2010 PMC3191757

[11] Orehek J, Charpin D, Velardocchio JM, Grimaud C (1980) Bronchomotor effect of bronchoconstriction-induced deep inspirations in asthmatics. Am Rev Respir Dis 121: 297–305. 7362137 10.1164/arrd.1980.121.2.297

[12] Lim TK, Pride NB, Ingram RH Jr (1987) Effects of volume history during spontaneous and acutely induced air-flow obstruction in asthma. Am Rev Respir Dis 135: 591–596. 3826885 10.1164/arrd.1987.135.3.591

[13] Faisy C, Pinto FM, Le Guen M, Naline E, Delyle SG, et al (2011) Airway response to acute mechanical stress in a human bronchial model of stretch. Crit Care 15: R208. 21914176 10.1186/cc10443 PMC3334752

[14] Liang H, Li WH (2009) Lowly expressed human microRNA genes evolve rapidly. Mol Biol Evol 26: 1195–1198. 19299536 10.1093/molbev/msp053 PMC2727378

[15] Kern HB, Niemeyer BF, Parrish JK, Kerr CA, Yaghi NK, et al (2012) Control of MicroRNA-21 expression in colorectal cancer cells by oncogenic epidermal growth factor/Ras signaling and Ets transcription factors. DNA Cell Biol 31: 1403–1411. 22553926 10.1089/dna.2011.1469 PMC3405454

[16] Moschos SA, Williams AE, Perry MM, Birrell MA, Belvisi MG, et al.. (2007) Expression profiling in vivo demonstrates rapid changes in lung microRNA levels following lipopolysaccharide-induced inflammation but not in the anti-inflammatory action of glucocorticoids. BMC Genomics 8. 10.1186/1471-2164-8-240 PMC1940008 17640343

[17] Yehya N, Yerrapureddy A, Tobias J, Margulies SS (2012) MicroRNA modulate alveolar epithelial response to cyclic stretch. BMC Genomics 13: 154. 22537220 10.1186/1471-2164-13-154 PMC3425319

[18] Caruso P, MacLean MR, Khanin R, McClure J, Soon E, et al (2010) Dynamic changes in lung microRNA profiles during the development of pulmonary hypertension due to chronic hypoxia and monocrotaline. Arterioscler Thromb Vasc Biol 30: 716–723. 20110569 10.1161/ATVBAHA.109.202028

[19] Ariel D, Upadhyay D (2012) The role and regulation of microRNAs in asthma. Curr Opin Allergy Clin Immunol 12: 49–52. 22157155 10.1097/ACI.0b013e32834ecb7f

[20] Oglesby IK, McElvaney NG, Greene CM (2010) MicroRNAs in inflammatory lung disease–master regulators or target practice? Respir Res 11: 148. 21029443 10.1186/1465-9921-11-148 PMC2984500

[21] Fulmer MT, Brown PW (1998) Hydrolysis of dicalcium phosphate dihydrate to hydroxyapatite. Journal of Materials Science-Materials in Medicine 9: 197–202. 15348892 10.1023/a:1008832006277

[22] Jiang YH, Jahagirdar BN, Reinhardt RL, Schwartz RE, Keene CD, et al (2002) Pluripotency of mesenchymal stem cells derived from adult marrow. Nature 418: 41–49. 12077603 10.1038/nature00870

[23] Pittenger MF, Mackay AM, Beck SC, Jaiswal RK, Douglas R, et al (1999) Multilineage potential of adult human mesenchymal stem cells. Science 284: 143–147. 10102814 10.1126/science.284.5411.143

[24] Al-Khaldi A, Eliopoulos N, Martineau D, Lejeune L, Lachapelle K, et al (2003) Postnatal bone marrow stromal cells elicit a potent VEGF-dependent neoangiogenic response in vivo. Gene Therapy 10: 621–629. 12692590 10.1038/sj.gt.3301934

[25] Lee KD, Kuo TKC, Whang-Peng J, Chung YF, Lin CT, et al (2004) In vitro hepatic differentiation of human mesenchymal stem cells. Hepatology 40: 1275–1284. 15562440 10.1002/hep.20469

[26] Liu G, Pareta RA, Wu R, Shi Y, Zhou X, et al.. (2012) Skeletal myogenic differentiation of urine-derived stem cells and angiogenesis using microbeads loaded with growth factors. Biomaterials. 10.1016/j.biomaterials.2012.10.038 PMC3513922 23137393

[27] Kuo TK, Ho JH, Lee OK (2009) Mesenchymal Stem Cell Therapy for Nonmusculoskeletal Diseases: Emerging Applications. Cell Transplantation 18: 1013–1028. 19523328 10.3727/096368909X471206

[28] Zou XH, Cai HX, Yin Z, Chen X, Jiang YZ, et al (2009) A Novel Strategy Incorporated the Power of Mesenchymal Stem Cells to Allografts for Segmental Bone Tissue Engineering. Cell Transplantation 18: 433–441. 19622230 10.3727/096368909788809839

[29] Waterman RS, Tomchuck SL, Henkle SL, Betancourt AM (2010) A new mesenchymal stem cell (MSC) paradigm: polarization into a pro-inflammatory MSC1 or an Immunosuppressive MSC2 phenotype. PLoS One 5: e10088. 20436665 10.1371/journal.pone.0010088 PMC2859930

[30] Ko IK, Kim BG, Awadallah A, Mikulan J, Lin P, et al (2010) Targeting improves MSC treatment of inflammatory bowel disease. Mol Ther 18: 1365–1372. 20389289 10.1038/mt.2010.54 PMC2911249

[31] Gervasio OL, Phillips WD, Cole L, Allen DG (2011) Caveolae respond to cell stretch and contribute to stretch-induced signaling. J Cell Sci 124: 3581–3590. 22045729 10.1242/jcs.084376

[32] Sotoudeh M, Jalali S, Usami S, Shyy JY, Chien S (1998) A strain device imposing dynamic and uniform equi-biaxial strain to cultured cells. Ann Biomed Eng 26: 181–189. 9525759 10.1114/1.88

[33] Fredberg JJ (2000) Frozen objects: small airways, big breaths, and asthma. J Allergy Clin Immunol 106: 615–624. 11031329 10.1067/mai.2000.109429

[34] Le Bellego F, Plante S, Chakir J, Hamid Q, Ludwig MS (2006) Differences in MAP kinase phosphorylation in response to mechanical strain in asthmatic fibroblasts. Respir Res 7: 68. 16643666 10.1186/1465-9921-7-68 PMC1459148

[35] Bonfield TL, Koloze M, Lennon DP, Zuchowski B, Yang SE, et al (2010) Human mesenchymal stem cells suppress chronic airway inflammation in the murine ovalbumin asthma model. Am J Physiol Lung Cell Mol Physiol 299: L760–770. 20817776 10.1152/ajplung.00182.2009 PMC4116401

[36] Cho JY, Pham A, Rosenthal P, Miller M, Doherty T, et al. Chronic OVA allergen challenged TNF p55/p75 receptor deficient mice have reduced airway remodeling. Int Immunopharmacol 11: 1038–1044. 10.1016/j.intimp.2011.02.024 PMC3121895 21382533

[37] Curley GF, Hayes M, Ansari B, Shaw G, Ryan A, et al. Mesenchymal stem cells enhance recovery and repair following ventilator-induced lung injury in the rat. Thorax. 10.1136/thoraxjnl-2011-201059 22106021

[38] Hammerschmidt S, Kuhn H, Grasenack T, Gessner C, Wirtz H (2004) Apoptosis and necrosis induced by cyclic mechanical stretching in alveolar type II cells. Am J Respir Cell Mol Biol 30: 396–402. 12959945 10.1165/rcmb.2003-0136OC

[39] Abdi R, Fiorina P, Adra CN, Atkinson M, Sayegh MH (2008) Immunomodulation by mesenchymal stem cells: a potential therapeutic strategy for type 1 diabetes. Diabetes 57: 1759–1767. 18586907 10.2337/db08-0180 PMC2453631

[40] Newman RE, Yoo D, LeRoux MA, Danilkovitch-Miagkova A (2009) Treatment of inflammatory diseases with mesenchymal stem cells. Inflamm Allergy Drug Targets 8: 110–123. 19530993 10.2174/187152809788462635

[41] Manning E, Pham S, Li S, Vazquez-Padron RI, Mathew J, et al (2010) Interleukin-10 Delivery via Mesenchymal Stem Cells: A Novel Gene Therapy Approach to Prevent Lung Ischemia-Reperfusion Injury. Human Gene Therapy 21: 713–727. 20102275 10.1089/hum.2009.147

[42] Pedersen IM, Otero D, Kao E, Miletic AV, Hother C, et al (2009) Onco-miR-155 targets SHIP1 to promote TNFalpha-dependent growth of B cell lymphomas. Embo Molecular Medicine 1: 288–295. 19890474 10.1002/emmm.200900028 PMC2771872

[43] O’Connell RM, Chaudhuri AA, Rao DS, Baltimore D (2009) Inositol phosphatase SHIP1 is a primary target of miR-155. Proc Natl Acad Sci U S A 106: 7113–7118. 19359473 10.1073/pnas.0902636106 PMC2678424

[44] Qi M, Elion EA (2005) MAP kinase pathways. J Cell Sci 118: 3569–3572. 16105880 10.1242/jcs.02470

[45] Qiu C, Feng MJ, Li FR, Chen CX, Lai YJ (2006) [The effect of phosphoinositide-3-kinase and signal transducer and activator of transcription-6 on the proliferation of T lymphocytes in bronchial asthma]. Zhonghua Jie He He Hu Xi Za Zhi 29: 688–693. 17129497

[46] Lee KS, Lee HK, Hayflick JS, Lee YC, Puri KD (2006) Inhibition of phosphoinositide 3-kinase delta attenuates allergic airway inflammation and hyperresponsiveness in murine asthma model. Faseb Journal 20: 455–465. 16507763 10.1096/fj.05-5045com

[47] Duan W, Aguinaldo Datiles AM, Leung BP, Vlahos CJ, Wong WS (2005) An anti-inflammatory role for a phosphoinositide 3-kinase inhibitor LY294002 in a mouse asthma model. International Immunopharmacology 5: 495–502. 15683846 10.1016/j.intimp.2004.10.015

[48] Kumar RK, Herbert C, Foster PS (2008) The “classical” ovalbumin challenge model of asthma in mice. Curr Drug Targets 9: 485–494. 18537587 10.2174/138945008784533561

[49] Min CK, Kim BG, Park G, Cho B, Oh IH (2007) IL-10-transduced bone marrow mesenchymal stem cells can attenuate the severity of acute graft-versus-host disease after experimental allogeneic stem cell transplantation. Bone Marrow Transplant 39: 637–645. 17369865 10.1038/sj.bmt.1705644

[50] Gupta N, Su X, Popov B, Lee JW, Serikov V, et al (2007) Intrapulmonary delivery of bone marrow-derived mesenchymal stem cells improves survival and attenuates endotoxin-induced acute lung injury in mice. J Immunol 179: 1855–1863. 17641052 10.4049/jimmunol.179.3.1855

[51] Gonzalez MA, Gonzalez-Rey E, Rico L, Buscher D, Delgado M (2009) Adipose-derived mesenchymal stem cells alleviate experimental colitis by inhibiting inflammatory and autoimmune responses. Gastroenterology 136: 978–989. 19135996 10.1053/j.gastro.2008.11.041

[52] Oh JY, Kim MK, Shin MS, Lee HJ, Ko JH, et al (2008) The anti-inflammatory and anti-angiogenic role of mesenchymal stem cells in corneal wound healing following chemical injury. Stem Cells 26: 1047–1055. 18192235 10.1634/stemcells.2007-0737

[53] Song G, Ju Y, Shen X, Luo Q, Shi Y, et al (2007) Mechanical stretch promotes proliferation of rat bone marrow mesenchymal stem cells. Colloids Surf B Biointerfaces 58: 271–277. 17499488 10.1016/j.colsurfb.2007.04.001

[54] Kasper G, Dankert N, Tuischer J, Hoeft M, Gaber T, et al (2007) Mesenchymal stem cells regulate angiogenesis according to their mechanical environment. Stem Cells 25: 903–910. 17218399 10.1634/stemcells.2006-0432

[55] Garbacki N, Di Valentin E, Huynh-Thu VA, Geurts P, Irrthum A, et al (2011) MicroRNAs profiling in murine models of acute and chronic asthma: a relationship with mRNAs targets. PLoS One 6: e16509. 21305051 10.1371/journal.pone.0016509 PMC3030602

[56] McCoy CE, Sheedy FJ, Qualls JE, Doyle SL, Quinn SR, et al (2010) IL-10 inhibits miR-155 induction by toll-like receptors. Journal of Biological Chemistry 285: 20492–20498. 20435894 10.1074/jbc.M110.102111 PMC2898307

[57] Yi R, Fuchs E (2011) MicroRNAs and their roles in mammalian stem cells. J Cell Sci 124: 1775–1783. 21576351 10.1242/jcs.069104 PMC3096054

[58] Lam AP, Dean DA (2008) Cyclic stretch-induced nuclear localization of transcription factors results in increased nuclear targeting of plasmids in alveolar epithelial cells. J Gene Med 10: 668–678. 18361478 10.1002/jgm.1187 PMC4084625

[59] Onyeagucha BC, Mercado-Pimentel ME, Hutchison J, Flemington EK, Nelson MA (2013) S100P/RAGE signaling regulates microRNA-155 expression via AP-1 activation in colon cancer. Exp Cell Res. 10.1016/j.yexcr.2013.05.009 PMC3726211 23693020

[60] Wehner S, Buchholz BM, Schuchtrup S, Rocke A, Schaefer N, et al (2010) Mechanical strain and TLR4 synergistically induce cell-specific inflammatory gene expression in intestinal smooth muscle cells and peritoneal macrophages. Am J Physiol Gastrointest Liver Physiol 299: G1187–1197. 20829523 10.1152/ajpgi.00452.2009

[61] Elton TS, Selemon H, Elton SM, Parinandi NL (2012) Regulation of the MIR155 host gene in physiological and pathological processes. Gene. 10.1016/j.gene.2012.12.009 23246696

[62] Tili E, Croce CM, Michaille JJ (2009) miR-155: on the crosstalk between inflammation and cancer. Int Rev Immunol 28: 264–284. 19811312 10.1080/08830180903093796

[63] Zhou T, Garcia JG, Zhang W (2011) Integrating microRNAs into a system biology approach to acute lung injury. Transl Res 157: 180–190. 21420028 10.1016/j.trsl.2011.01.010 PMC3073780

[64] Li LF, Ouyang B, Choukroun G, Matyal R, Mascarenhas M, et al (2003) Stretch-induced IL-8 depends on c-Jun NH2-terminal and nuclear factor-kappaB-inducing kinases. Am J Physiol Lung Cell Mol Physiol 285: L464–475. 12716652 10.1152/ajplung.00031.2003

[65] Bhattacharyya S, Balakathiresan NS, Dalgard C, Gutti U, Armistead D, et al (2011) Elevated miR-155 promotes inflammation in cystic fibrosis by driving hyperexpression of interleukin-8. J Biol Chem 286: 11604–11615. 21282106 10.1074/jbc.M110.198390 PMC3064214

