# Supplementary figures and images for: Human Mesenchymal Stem Cells Suppress the Stretch–Induced Inflammatory miR-155 and Cytokines in Bronchial Epithelial Cells
Source: PLoS One. 2013 Aug 13;8(8):e71342. doi: 10.1371/journal.pone.0071342 (PMC3742760; doi:10.1371/journal.pone.0071342)

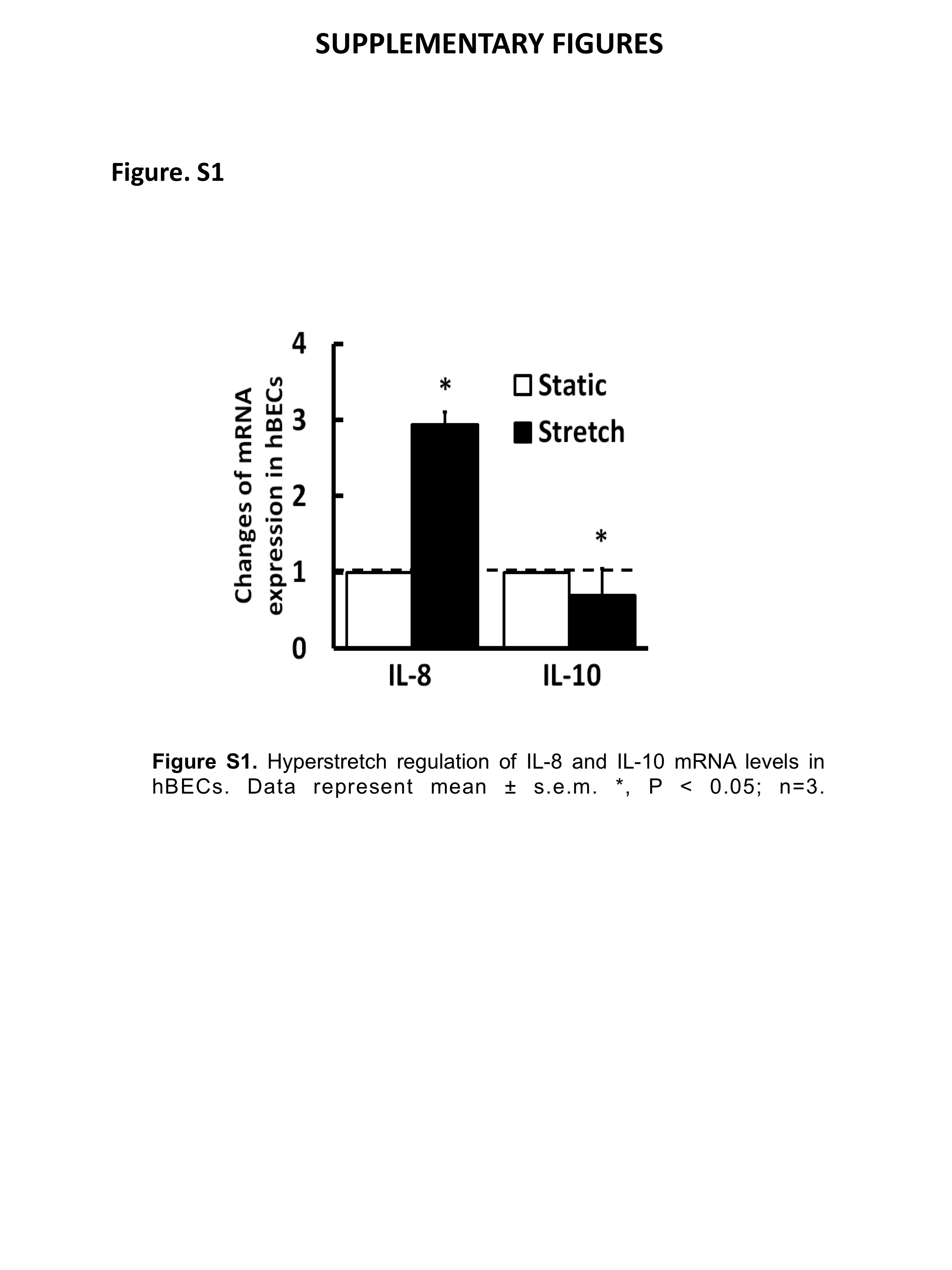

Supplement: Figure S1 — Hyperstretch regulation of IL-8 and IL-10 mRNA levels in hBECs. Data represent mean ± s.e.m. *, P<0.05; n = 3. (TIF) [file pone.0071342.s001.tif]

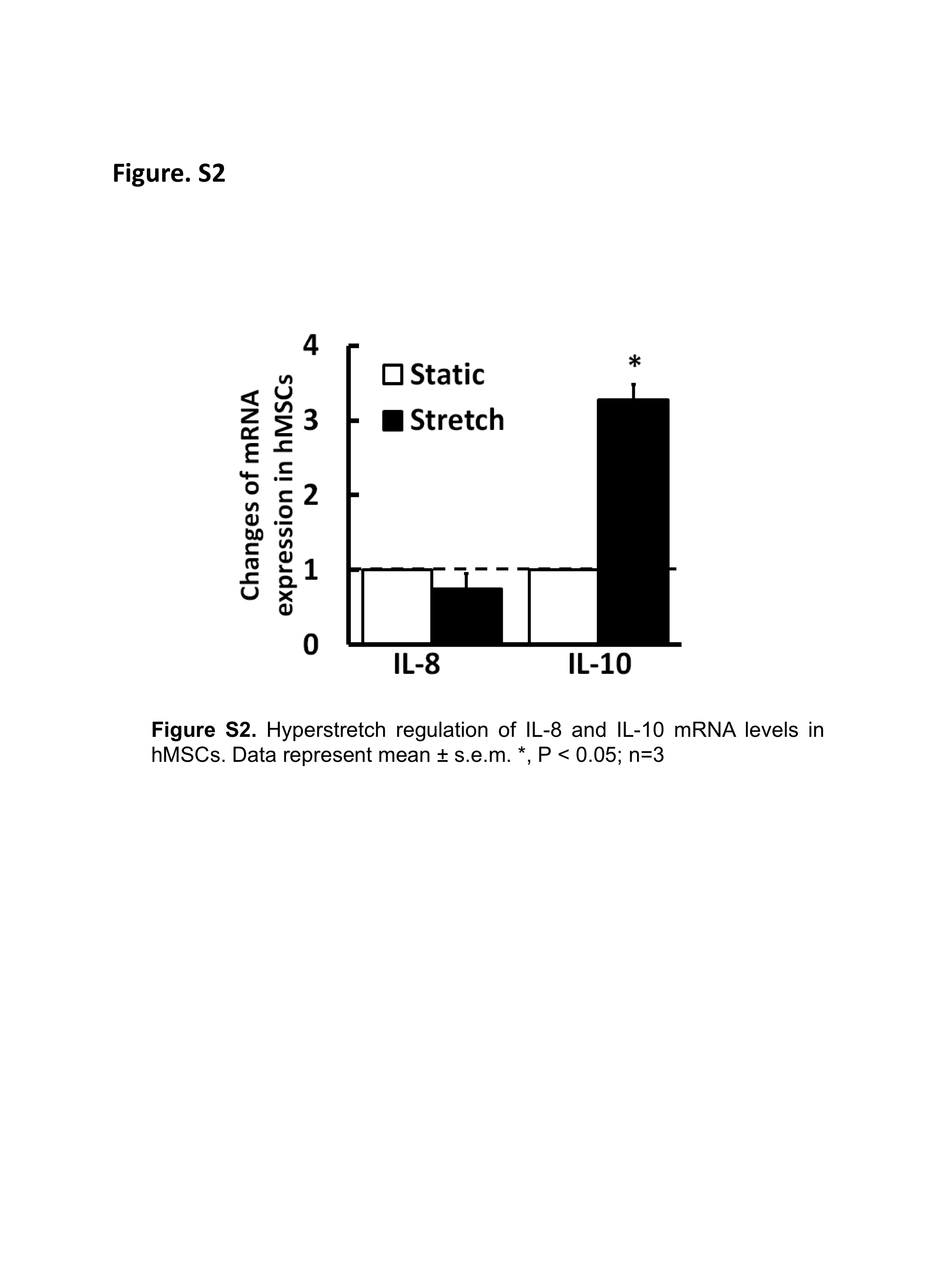

Supplement: Figure S2 — Hyperstretch regulation of IL-8 and IL-10 mRNA levels in hMSCs. Data represent mean ± s.e.m. *, P<0.05; n = 3. (TIF) [file pone.0071342.s002.tif]

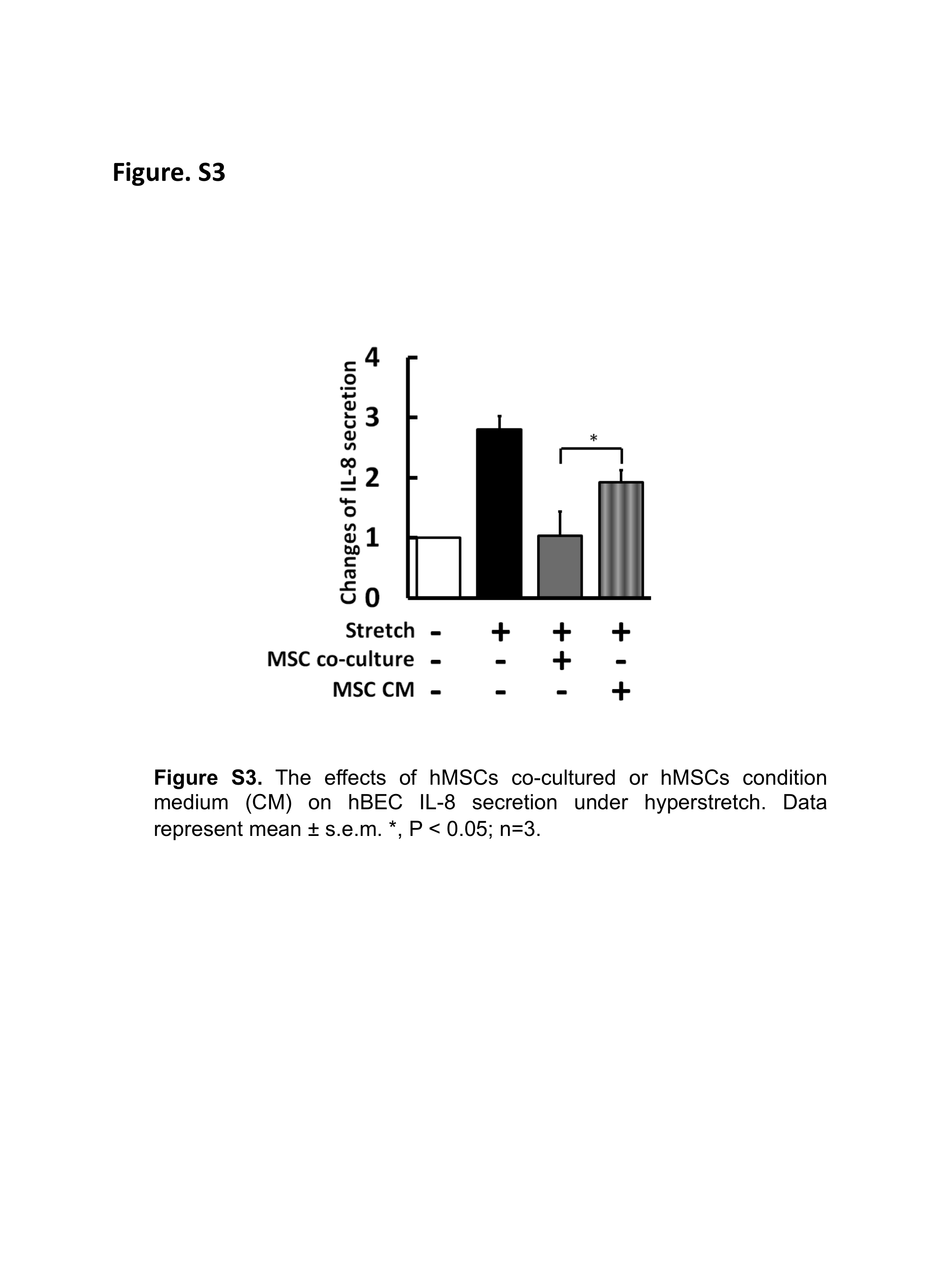

Supplement: Figure S3 — The effects of hMSCs co-cultured or hMSCs condition medium (CM) on hBEC IL-8 secretion under hyperstretch. Data represent mean ± s.e.m. *, P<0.05; n = 3. (TIF) [file pone.0071342.s003.tif]

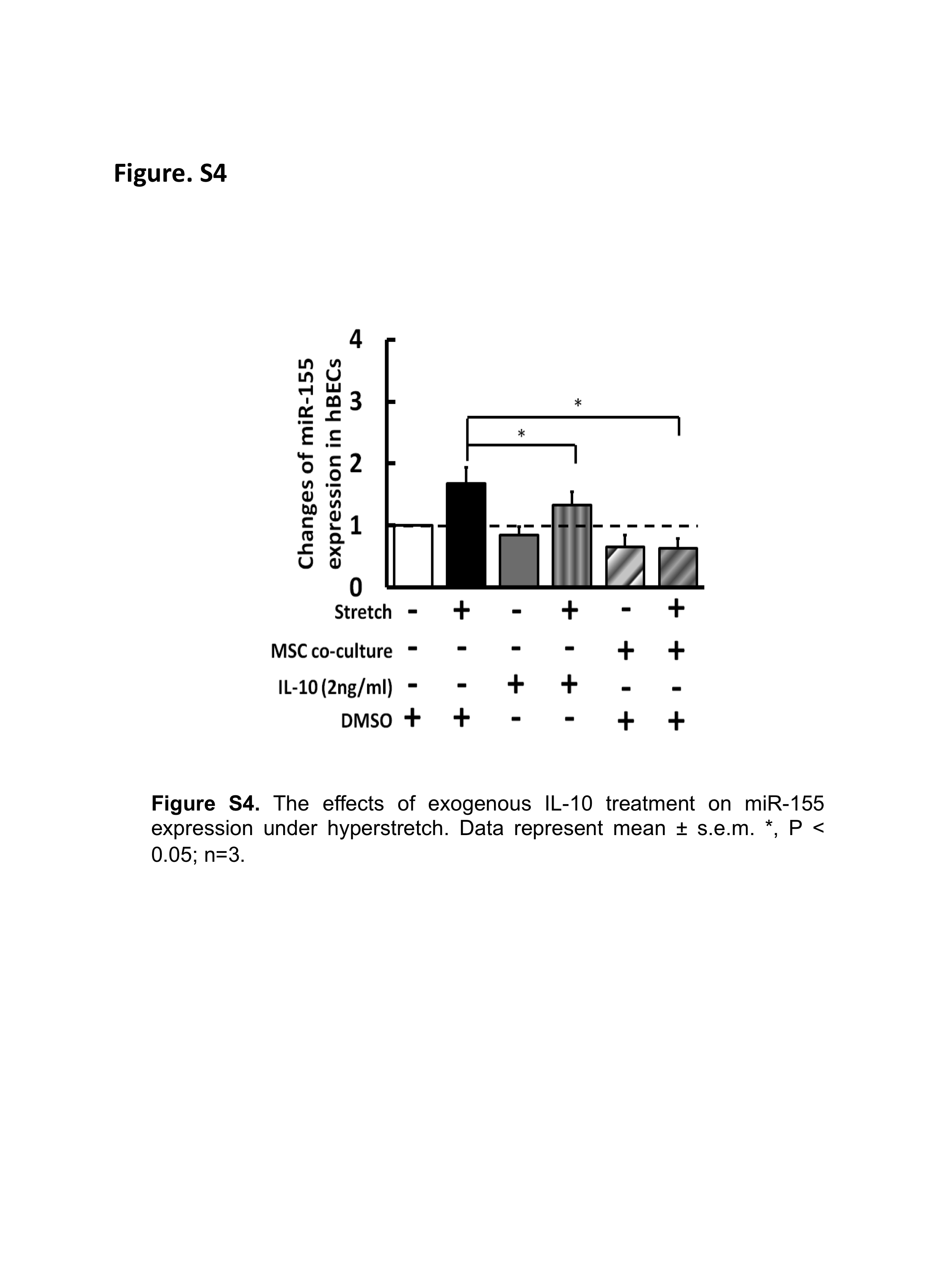

Supplement: Figure S4 — The effects of exogenous IL-10 treatment on miR-155 expression under hyperstretch. Data represent mean ± s.e.m. *, P<0.05; n = 3. (TIF) [file pone.0071342.s004.tif]

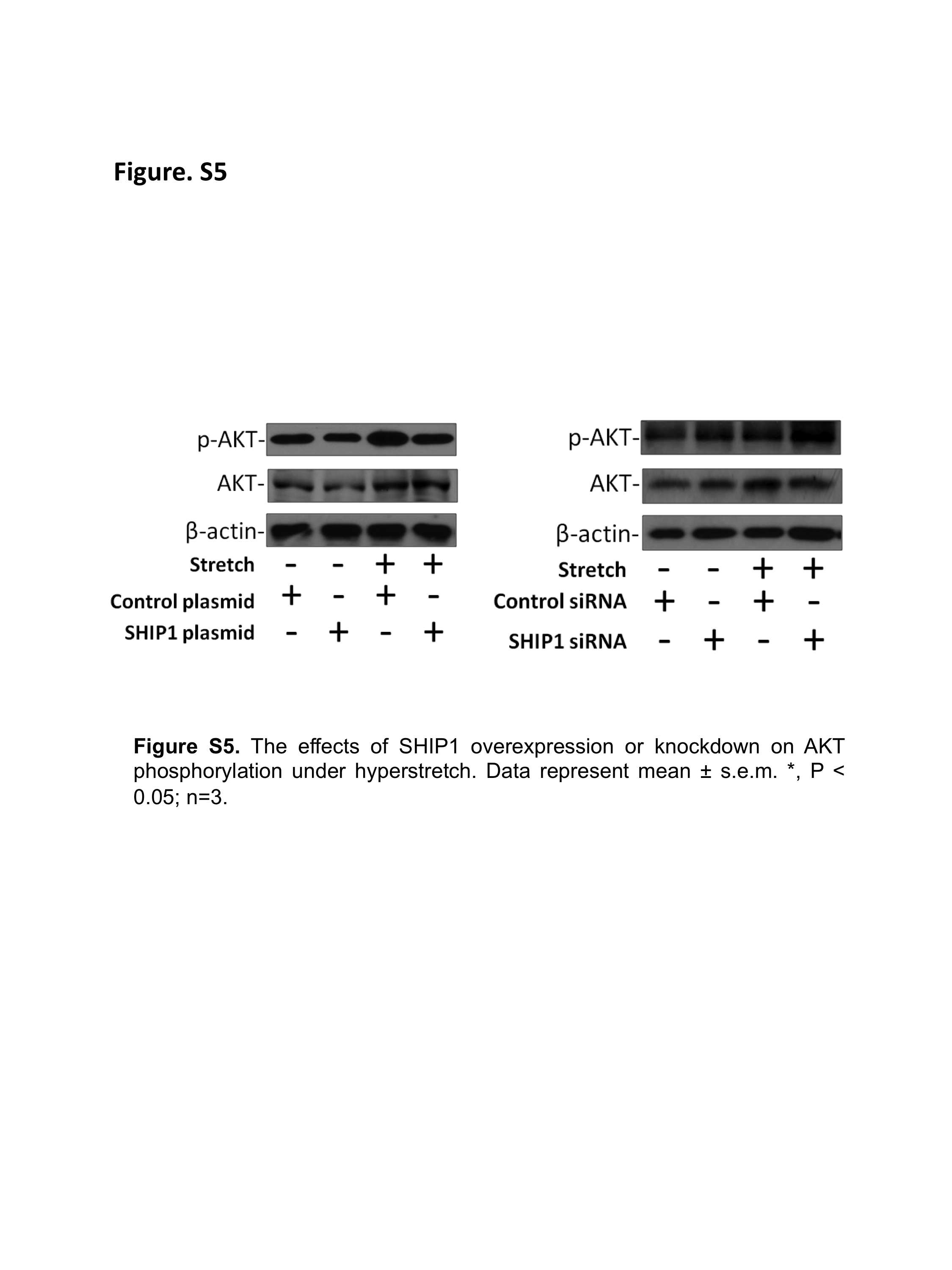

Supplement: Figure S5 — The effects of SHIP1 overexpression or knockdown on AKT phosphorylation under hyperstretch. Data represent mean ± s.e.m. *, P<0.05; n = 3. (TIF) [file pone.0071342.s005.tif]

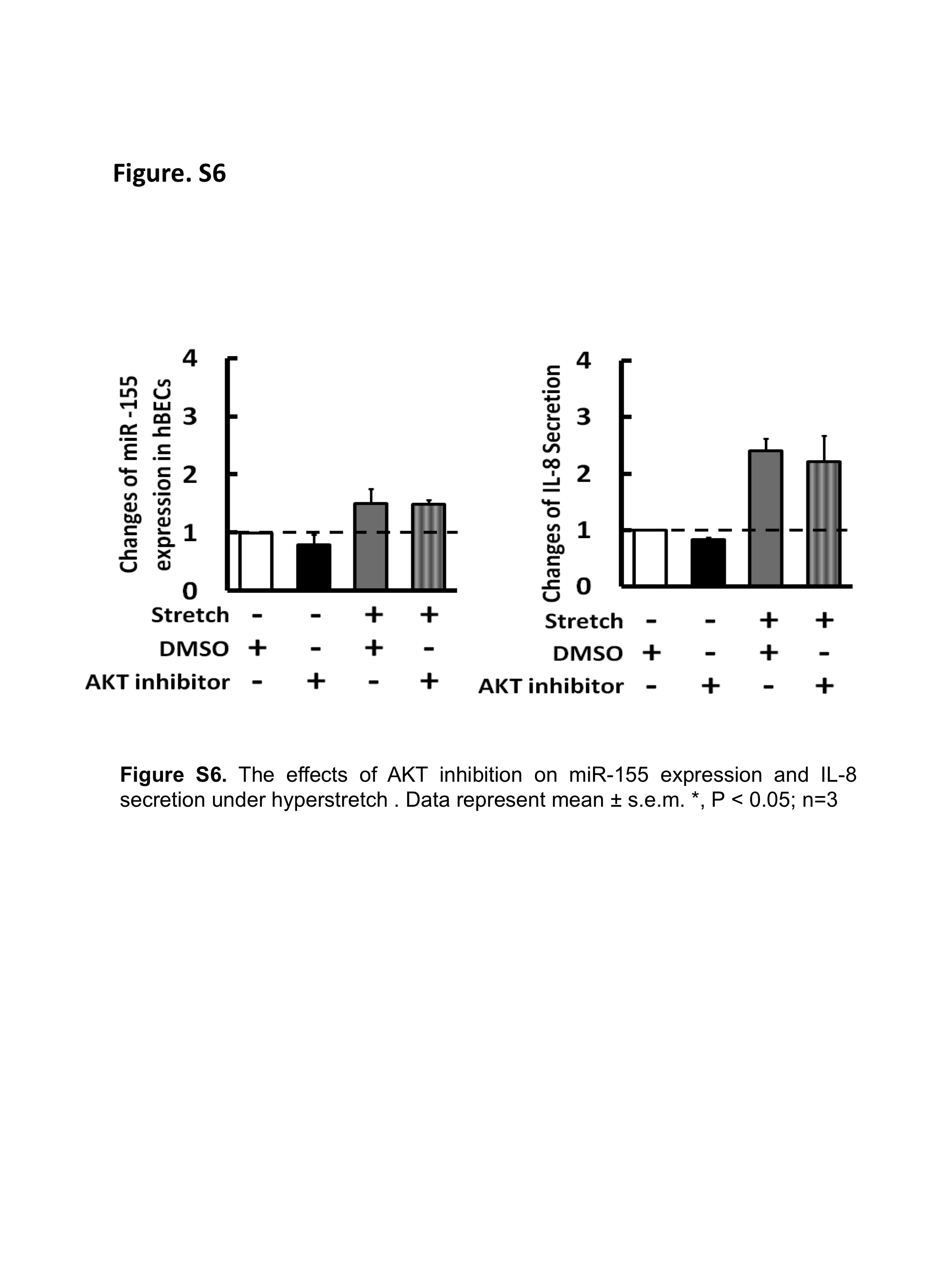

Supplement: Figure S6 — The effects of AKT inhibition on miR-155 expression and IL-8 secretion under hyperstretch. Data represent mean ± s.e.m. *, P<0.05; n = 3. (TIF) [file pone.0071342.s006.tif]
